# Loss of αklotho causes reduced motor ability and short lifespan in zebrafish

**DOI:** 10.1038/s41598-021-93909-y

**Published:** 2021-07-23

**Authors:** Yurie Ogura, Ryoji Kaneko, Kota Ujibe, Yuma Wakamatsu, Hiromi Hirata

**Affiliations:** Department of Chemistry and Biological Science, College of Science and Engineering, Aoyama Gakuin University, Sagamihara, 252-5258 Japan

**Keywords:** Developmental biology, Disease model

## Abstract

The *klotho* gene encodes a transmembrane protein αKlotho that interacts with a fibroblast growth factor (FGF) receptor in renal tubular epithelial cells and functions as a co-receptor for FGF23, which is an osteocytes-derived hormone. This bone-to-kidney signal promotes urinary phosphate excretion. Interestingly, αKlotho knockout mice show an accelerated aging and a shortened life span. Similarly, *C. elegans* lacking the αklotho homologue showed a short life span. However, the physiological basis of aging-related function of αklotho remain unclear. The αklotho-deficient vertebrate animals other than mice have been awaited as an alternative model of premature aging. We here employed zebrafish in our study and revealed that αklotho mutant zebrafish appeared to be normal at 3 months postfertilization (mpf) but eventually underwent premature death by 9 mpf, while normal zebrafish is known to survive for 42 months. We also assessed the motor ability of zebrafish in a forced swimming assay and found that αklotho mutant zebrafish displayed reduced swimming performance before their survival declined. A recent study also reported a similar finding that αklotho-deficient zebrafish exhibited a short life span and reduced spontaneous movements. Taken together, these results suggest that αKlotho mutant zebrafish show premature aging and are useful to investigate aging in vertebrates.

## Introduction

Senescence is a process of cell cycle arrest, while aging is a process of gradual deterioration of tissue functions. In human aging, a number of physiological decline of tissue functions occur over time, accompanying the increase of skin wrinkling, soft tissue calcification, neural degeneration, muscle weakness and motor deterioration^1^. Numerous aging-related disorders and premature aging diseases have been pathologically identified in human. Although it has been suggested that aging is triggered by an accumulation of DNA/cell damage or by a genetic limitation of cell proliferation, the physiological basis and causes of aging are still largely unknown^1^. To investigate aging-related genes and to fight against aging, several animal models such as mice, fruit flies and *C. elegans* have been used to assay longevity and premature death^2–5^. Zebrafish (*Danio rerio*), which is an emerging alternative vertebrate model, has also been used to study the progressive deterioration of biological functions in aging^6, 7^.

The *klotho* (*kl*) gene was originally identified as an aging-suppressor in mutant mice that showed an accelerated aging and a short lifespan^8^. The *kl* gene encodes a single-pass transmembrane protein αklotho and is predominantly expressed in the distal convoluted tubules. The αklotho protein binds to fibroblast growth factor (FGF) receptors and functions as a co-receptor for FGF23, which is secreted from osteocytes in bone^9^. This bone-to-kidney signal is activated by an increase of serum phosphorus, promoting phosphate discharge from blood to urine^10^. The αklotho-deficient mice as well as FGF23 knockout mice exhibited an increase of blood phosphate that resulted in arteriosclerosis and vascular calcification^11, 12^. In addition to these blood vessel disorders, multiple aging phenotypes such as skin atrophy, auditory disturbance, osteopenia, sarcopenia and premature death has been reported in these mutant mice^13^. Conversely, transgenic mice that overexpress αklotho displayed longevity compared to normal mice^14^. Thus, one important contributing factor to αklotho-mediated suppression of aging appears to be the regulation of phosphate levels. But how phosphate homeostasis determines the life span remains unclear^15^. The αklotho-deficient vertebrate animals other than mice would contribute toward understanding aging.

Here, we employed zebrafish in our αklotho study and found that αklotho mutant zebrafish (Y306X) showed a shortened lifespan. A recent study reported the same finding that αklotho-deficient zebrafish (S179frameshift) underwent a premature death^16^. We also assessed swimming performance of zebrafish in a forced swimming assay and demonstrated that αklotho mutant zebrafish display a reduced swimming ability before their survival declined.

## Results

### Zebrafish *klotho* gene and *klotho* mutation

To study αklotho in zebrafish, we first retrieved *klotho* gene information of zebrafish and the other vertebrate animals from NCBI database. An amino acid alignment of the αklotho showed that αklotho protein is conserved among vertebrates from zebrafish to human, especially in two glycosidase domains (Supplemental Fig. 1). Although amino acid residues of the transmembrane domain at the C-terminus of the αklotho appeared to be less conserved in chicken, frog and zebrafish, online prediction tools of the protein secondary structure such as PredictProtein and SOSUI suggested that this region is a putative membrane-spanning domain in these non-mammalian animals, verifying that αklotho is overall conserved among vertebrates.

**Figure 1:**
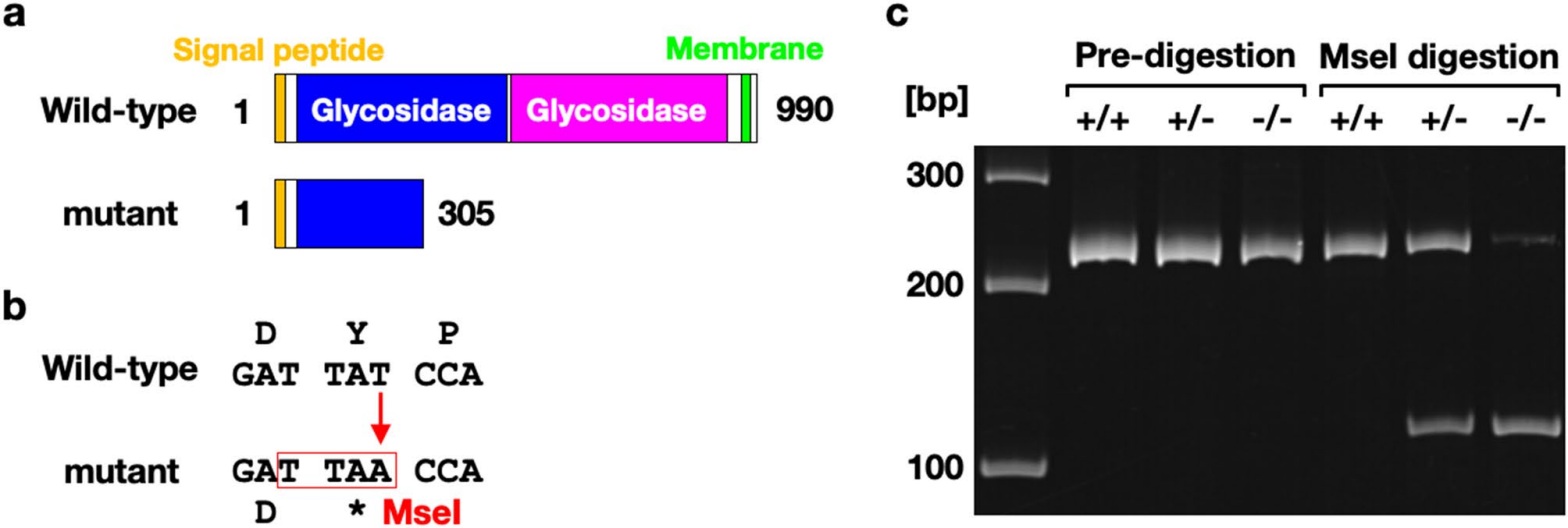
A nonsense mutation of zebrafish *kl gene*. (**a**) The αklotho protein has a signal peptide, two glycosidase domains and a transmembrane domain. A nonsense mutation caused a truncation in the first glycosidase domain. (**b**) The T-to-A mutation generated an MseI restriction enzyme site. (**c**) The size of the genomic PCR products was 240 bp. The PCR products amplified from mutant alleles were digested by MseI to generate two 120-bp fragments.

To investigate the physiological function of αklotho in zebrafish, we next obtained an αklotho mutant allele *kl*^*sa18644*^, which was identified in a targeting induced local lesions in genomes (TILLING) project^17^. This allele harbors a T to A base substitution that generates a premature nonsense codon (Y306X) in the middle of the first glycosidase domain (Fig. 1a). This mutation also generated an MseI restriction site that enabled genotyping by a restriction enzyme digestion of the genomic PCR products (Fig. 1b,c).

### Zebrafish *klotho* mutants exhibit short life spans

**Figure 2:**
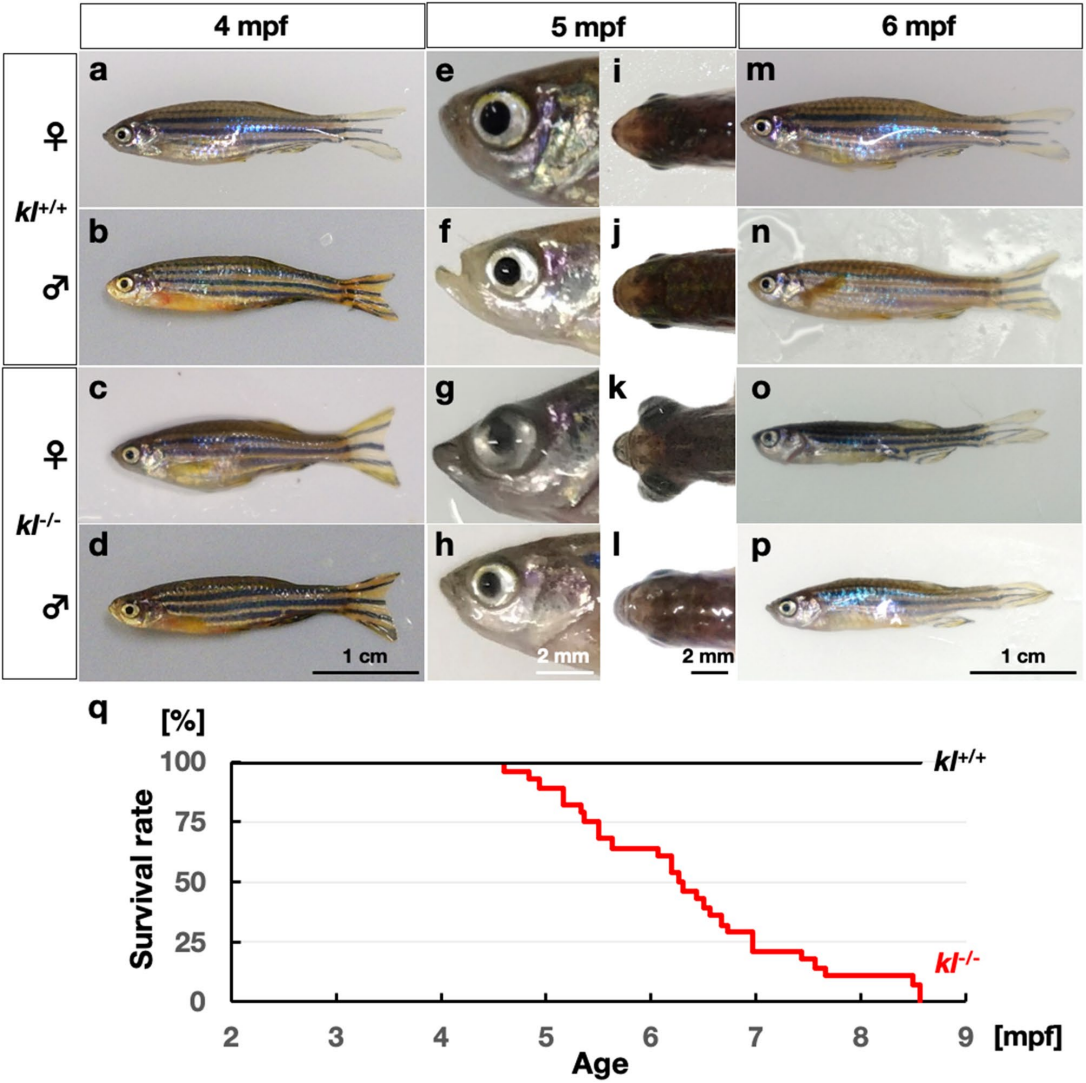
Zebrafish *kl* mutants become thinner and undergo premature death. (**a**–**d**) The *kl*^+/+^ female (**a**), *kl*^+/+^ male (**b**), *kl*^−/−^ female (**c**) and *kl*^−/−^ male (**d**) zebrafish at 4 mpf. The *kl*^−/−^ fish showed no morphological defects at this stage. (**e**–**l**) Lateral and dorsal views of head in *kl*^+/+^ female (**e**,**i**), *kl*^+/+^ male (**f**,**j**), *kl*^−/−^ female (**g**,**k**) and *kl*^−/−^ male (**h**,**l**) at 5 mpf. Note that the eyeball is protruding to the outside in the *kl*^−/−^ female. (**m**–**p**) The *kl*^+/+^ female (**m**), *kl*^+/+^ male (**n**), *kl*^−/−^ female (**o**) and *kl*^−/−^ male (**p**) zebrafish at 6 mpf. The *kl*^−/−^ fish became skinny. (**q**) The *kl*^+/+^ fish (n = 32) survived over 9 mpf. All of the *kl*^−/−^ fish (n = 28) underwent premature death before 9 mpf.

By crossing heterozygous *kl* mutant carrier fish, we obtained homozygous *kl* mutants (*kl*^−/−^). The *kl*^−/−^ embryos showed no apparent defects and grew up to become adults. The ratio of *kl*^−/−^ mutants in a progeny of a heterozygous carrier cross was about one quarter (23%, 23/100) at 4 months postfertilization (mpf), indicating that the loss of *kl* does not affect development or survival of zebrafish in both females and males until they become young adults (Fig. 2a–d) as reported recently^16^. Although the skin of the previously reported *kl*-deficient zebrafish appeared to be pale at 5 mpf^16^, we did not see apparent reduction of the skin tone in *kl*^−/−^ compared to *kl*^+/+^ fish at any age. Intriguingly, we noticed that some mutant female displayed protruding eye at 5 mpf (30%, 6/20) just like a telescope goldfish (Fig. 2e–l). This malformation of the eye was not seen in mutant male fish (n = 30). We also recapitulated that our *kl*^−/−^ fish become thinner after 5 months of age and die within 9 months (Fig. 2m–p). To detail the short life span of *kl*^−/−^ zebrafish, we monitored the survival of our *kl*^+/+^ and *kl*^−/−^ fish from 2 mpf (Fig. 2q). We found that all of the *kl*^−/−^ fish (n = 28) died between 4 and 9 mpf, whereas all *kl*^+/+^ fish (n = 32) kept living after 9 mpf. It has been reported that the mean life span of zebrafish is 42 months^18^. These results indicate that *kl*^−/−^ zebrafish undergo premature aging and death.

### Zebrafish *klotho* mutants show reduced motor ability

Since aging affects the motor system^19^, we addressed whether motor ability is impaired in *kl*^−/−^ zebrafish. As swimming ability of adult zebrafish is governed by the body size especially by the caudal fin length^20^, we began with a physical measurement. We measured the standard length and caudal fin length, which are defined as the length from the head to the root of the caudal fin and from the root of the caudal fin to the edge of the fin, respectively^21^. Both standard length and caudal fin length of *kl*^+/+^ and *kl*^−/−^ fish were comparable in both females and males at 3 mpf and 4 mpf. These data confirm that *kl*^−/−^ fish are indistinguishable from *kl*^+/+^ fish by appearance at both 3 mpf and 4 mpf.

**Figure 3:**
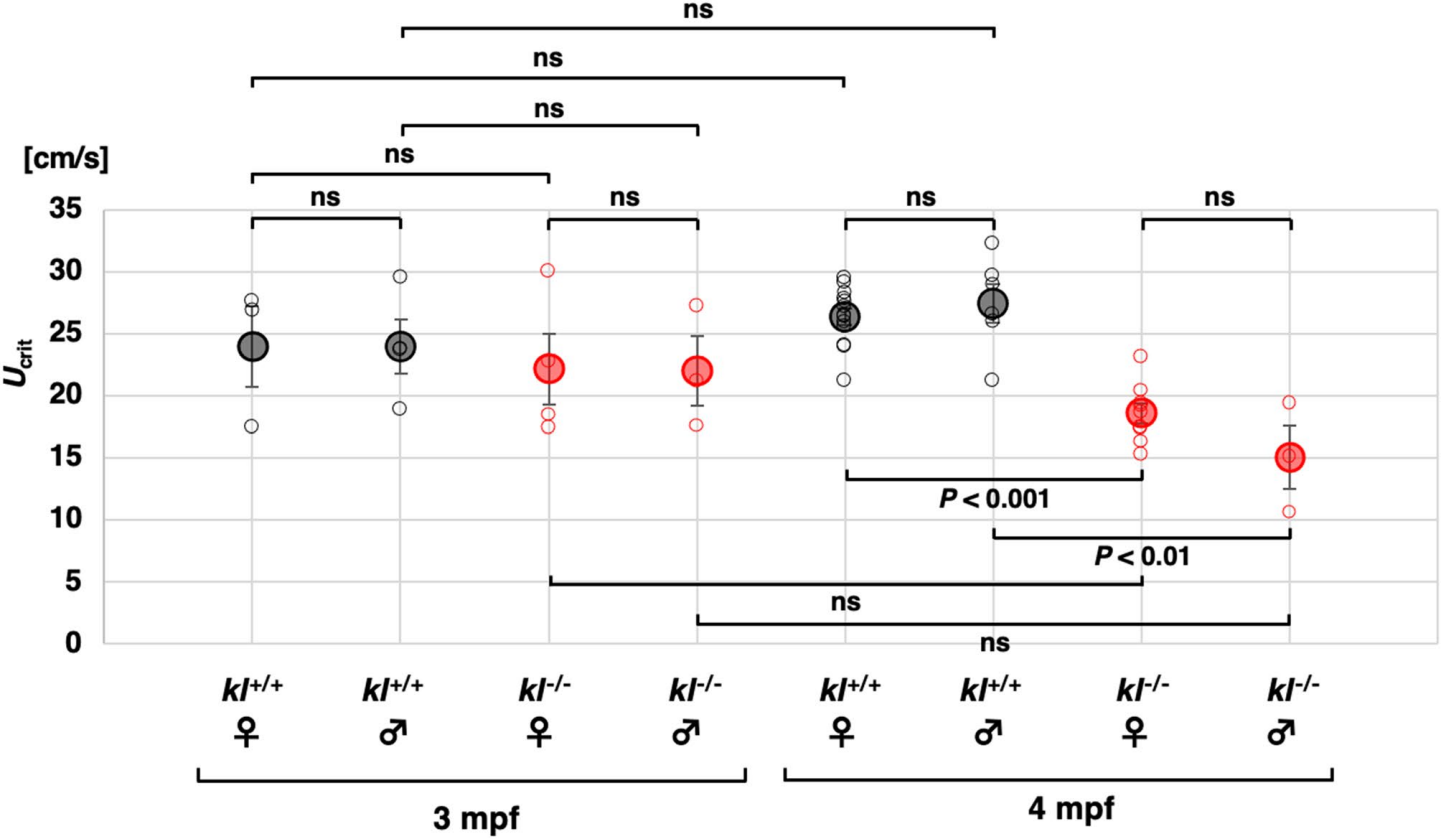
Swimming performance in *kl* mutants declines by 4 mpf. The critical swimming speed (*U*_crit_) of 3 mpf and 4 mpf zebrafish were measured using a swimmill. The *kl*^+/+^ female (n = 3), *kl*^+/+^ male (n = 4), *kl*^−/−^ female (n = 4) and *kl*^−/−^ male (n = 3) were assayed at 3 mpf. The *kl*^+/+^ female (n = 13), *kl*^+/+^ male (n = 9), *kl*^−/−^ female (n = 6) and *kl*^−/−^ male (n = 3) were assayed at 4 mpf. Open black (*kl*^+/+^) and red (*kl*^−/−^) circles indicate *U*_crit_ of individual samples. Closed black (*kl*^+/+^) and red (*kl*^−/−^) circles with error bars indicate mean ± sem.

To quantify the swimming performance of adult zebrafish, we then employed a swimmill, which is a treadmill for aquatic animals^20^. A zebrafish was put in a swimming chamber, where water flow is generated by a voltage-controlled spinning propeller. Since fish swim against current to maintain the position in the water flow, zebrafish in the chamber swim at the water velocity. The water velocity was initially set to 10 cm/s for 1 min and successively set to 15 cm/s for 1 min. Eventually, the water velocity increased 1 cm/s every 1 min and the water velocity at when zebrafish could no longer keep swimming was defined as the critical swimming speed (*U*_crit_) as described previously^22^. At 3 mpf, the *U*_crit_ of *kl*^−/−^ zebrafish (female 22.2 ± 2.9 cm/s, n = 4; male 22.0 ± 2.8 cm/s, n = 3; Fig. 3) were comparable to those of *kl*^+/+^ fish (female 24.0 ± 3.3 cm/s, n = 3, *P* = 0.70; male 24.0 ± 2.2 cm/s, n = 4; *P* = 0.59). At 4 mpf, however, the *U*_crit_ of *kl*^−/−^ zebrafish (female 18.6 ± 0.8 cm/s, n = 9; male 15.0 ± 2.5 cm/s, n = 3) were significantly lower than those of *kl*^+/+^ fish (female 26.4 ± 0.6 cm/s, n = 13, *P* < 0.001; male 27.4 ± 1.6 cm/s, n = 6; *P* < 0.01). These results demonstrate that the swimming performance of *kl*^−/−^ fish starts to decline between 3 and 4 mpf when they do not exhibit premature death.

## Discussion

In this study, we investigated a nonsense mutant allele of *kl* in zebrafish and found that *kl*^−/−^ fish showed a shortened life span. Recently, Singh and his colleagues have reported the other *kl* mutant zebrafish allele, which harbors a CRISPR-mediated 5-bp deletion and thus creates a frameshift in the first glycosidase domain^16^. Although age-related frailty and premature death in our *kl*^−/−^ fish were recapitulations of a recent report, we uniquely examined swimming performance of *kl*^−/−^ fish using a swimmill and demonstrated that *kl*^−/−^ fish initially exhibited comparable motor performance with *kl*^+/+^ fish at 3 mpf and eventually showed a compromised swimming at 4 mpf before they become thinner and undergo premature death at 5–8 mpf. Taken together, we conclude that αklotho mutant zebrafish show premature aging phenotypes and that αklotho-deficient zebrafish can be an animal model of progeria.

### Conservation of the αklotho in vertebrates

While invertebrate animals use calcium carbohydrate for exoskeleton, vertebrate animals utilize calcium phosphate for endoskeleton but with a risk of phosphate toxicity^23^. Since αklotho plays an essential role in discharging excess phosphate from blood to prevent unwanted ectopic calcification, it is reasonable that αklotho function is conserved among vertebrates^24^. Our amino acid alignment revealed that αklotho proteins have two putative glycosidase domains that are highly conserved among vertebrates. These domains share the highest homology with lactase, which hydrolyses lactose to produce galactose and glucose^25, 26^. Interestingly however, the catalytic glutamate residues in both glycosidase domains in αklotho were substituted to the other residues; i.e. N239 and S872 in human, N241 and A874 in mice and N208 and A849 in zebrafish. Instead of losing glycosidase activities, these domains appeared to acquire an affinity to a calciotropic hormone FGF23, enabling αklotho to function as a co-receptor of FGF23 for the excretion of phosphate from renal tubules^9^. These amino acid substitution might have been critical for αklotho function in vertebrates. An αklotho homologue gene (*klo1*) was also found in *C. elegans*, and *klo1*-deficient worms showed short life spans^27, 28^. The αklotho protein in *C. elegans* has only one glycosidase domain with its catalytic residue intriguingly maintained as glutamate, potentially possessing the glycosidase activity. But how αklotho-mediated suppression of aging takes place in *C. elegans* remains unsolved. Our *kl* mutant allele harbored a nonsense mutation in the first putative glycosidase domain, while the other allele reported by Singh et al. carried a frameshift mutation in the same glycosidase domain^16^. Both of these mutant alleles appear to disrupt the putative glycosidase domain and eliminate the transmembrane domain at the C-terminus and thus are likely unfunctional as a co-receptor for FGF23.

### Telescope eye phenotype in *kl*^−/−^ female zebrafish

The telescope eye goldfish strain was established 300 years ago and maintained in East Asia^29^. This developmental malformation of the eye is inherited to both female and male offspring in a recessive manner. A recent study revealed that nonsense mutations or a retrotransposon insertion in goldfish *lrp2al* gene, which encodes a low density lipoprotein receptor-related protein, was responsible for the telescope eye^30^. Likewise, the *lrp2a* mutation in zebrafish caused the protruding eye phenotype, which is often referred to as bugeye, in both female and male individuals with a variable penetration rate^31–33^. In our zebrafish *kl*^−/−^ case, telescope eye phenotype was seen in 30% of *kl*^−/−^ female but not in any *kl*^−/−^ male. Similarly, *fgf23*-deficient zebrafish exhibited the protruding eye only in the female^16^. Collectively, the αklotho-FGF23 signals may potentially regulate the expression or function of *lrp2a* in cooperation with the female-specific endocrine regulation, thereby affecting the eye development in female.

### Decline of motor performance in *kl*^−/−^ zebrafish

Previous in situ hybridization analyses revealed that *kl* gene is expressed by the brain, pancreas, liver and pronephros in embryonic and larval zebrafish and by the liver and mesonephric kidney tubules in adult zebrafish^34^. The *kl*^−/−^ zebrafish did not show any apparent defects during development and in young adults. But they eventually showed the decline of motor ability at 4 mpf, became thinner at 6 mpf and underwent premature death by 9 mpf. Although mutant phenotypes in zebrafish larvae can be generally rescued by mRNA injection-mediated overexpression of the gene product, this does not work in *kl*^−/−^ zebrafish, because all of the defects in *kl*^−/−^ were adult onset.

Since *kl* gene is expressed only by the liver and kidney in adult zebrafish^34^, the motor impairment, which is attributable to the neuronal dysfunction, muscle weakness or cardiovascular defects, could be a secondary phenotype caused through a perturbation of the endocrine system. Our future histological and physiological studies of neurons and muscles as well as metabolomic analyses at 3–4 mpf will clarify the basis of the motor deterioration. In our forced swimming assay, the decline of the motor ability was evaluated by the maximum swimming speed *U*_crit_. It has been reported that the reduction of *U*_crit_ accompanies with aging in zebrafish^35^, implying that motor deterioration in *kl*^−/−^ fish is a premature aging phenotype. Taken together, the decline of motor performance is likely one of the earliest physiological phenotype of premature aging in *kl*^−/−^ zebrafish.

### Pathology of αklotho

Kuro-o and his colleagues have demonstrated that αklotho mutant mice showed cellular senescence such as the upregulation of a cyclin-dependent kinase inhibitor p21 and cell cycle arrest as well as age-related disorders including the ectopic calcification and hypokinesis^8^. The life span of the mutant mice was approximately 60 days with no mice surviving over 100 days. A population-based association study suggested that a SNP that generates an F352V missense variant in human αklotho is implicated in a short life span^36^. In addition, a 13-year-old girl who carries a homozygous H193R missense variant in αklotho showed premature tumoral calcinosis with dural and carotid artery calcifications^37^. Thus, physiological symptoms linked to αklotho mutations appear to be common in vertebrates. Although pathological studies of αklotho deficiency has only been done in human, mice and *C. elegans* until recently, the latest publication of αklotho mutant zebrafish^16^ and our current study demonstrated pathologically-relevant progressive motor deterioration and premature death in zebrafish *kl* mutants. Collectively, zebrafish αklotho mutants provide an alternative animal model to study cellular senescence, aging and progeria in vertebrates.

## Materials and methods

### Animals

Zebrafish (*Danio rerio*) were reared and maintained in 1.7 L tanks in a recirculating Meito System (Meito System) under a 14 h light and 10 h dark photoperiod according to the standard protocol^38^. Larvae were fed paramecia and Gemma Micro ZF 75 (Funakoshi) twice a day from 5 days postfertilization to 1 mpf. Juvenile fish (1–3 mpf) were fed brine shrimp (Tokai Guppy) and Gemma Micro ZF 75 twice a day. Adults fish (3 mpf–) were fed brine shrimp and Otohime B2 (Marubeni Nissin Feed) twice a day. Zebrafish αklotho mutant line (*kl*^*sa18644*^) and wild-type AB line, the latter for the line maintenance, was purchased from Zebrafish International Resource Center (https://zebrafish.org/home/guide.php).

### Genotyping

The missense region of *kl* gene was amplified by genomic PCR using KAPA Taq Extra PCR Kit (Kapa Biosystems) in ProFlex PCR System (Thermo Fisher Scientific). Following program was used for amplification: 94 °C 2 min; 94 °C 10 s, 63 °C 20 s, 72 °C 30 s, 35 cycles; 72 °C 1 min; 4 °C forever. Following primers were used; *kl* genotyping forward: CTCTGGGATCTCACTGGATC; *kl* genotyping reverse: AACTAAGAGCAGGTCCATGAGAC. PCR products were digested with MseI restriction enzyme (Takara) and separated by 15% polyacrylamide gel electrophoresis at 300 V for 90 min as described previously^39^. The gel images were captured using the Printgraph AE-6933FXCF (Atto).

### Image capture

Zebrafish were anesthetized in 0.004% Tricaine (MS-222, Sigma-Aldrich) for 1 min. Images of zebrafish were captured using a digital camera SONY α5000.

### Survival

Adult zebrafish obtained by a cross of *kl*^+/−^ fish were maintained in the regular care. Genotyping was done at 2 mpf. Zebrafish (*kl*^+/+^ and *kl*^−/−^) that kept floating at water surface or sinking at the bottom without swimming for 1 min were judged as reaching the end point and subjected to euthanasia and genotyping. Number of zebrafish reaching the end point was counted everyday until all of the *kl*^−/−^ fish died.

### Swimmill analysis

Motor ability of zebrafish was quantified using a swimmill system Swim tunnel respirometer 170 ml (Loligo System) as described previously^20^. In brief, an adult zebrafish, which was kept unfed for 24 h, was put under a propeller-driven water flow in a chamber, and fish was compelled to swim in the water flow. The water velocity increased 1 cm/s every 1 min after initial warming up of 10 cm/s flow for 1 min and successive 15 cm/s flow for 1 min. Zebrafish swam at the speed of water flow until the water velocity reach the maximum swimming capability of the fish. The water velocity at when zebrafish can no longer keep swimming was defined as critical swimming speed *U*_crit_. The standard and caudal fin lengths were measured by analyzing frames of swimming movies as described previously^20^.

### Statistics

The quantitative data were given as mean ± sem. All error bars in graphs indicate the sem. The sample numbers are shown in Figure legends. The quantitative data were tested for normality by Shapiro–Wilk test (P < 0.05). Statistical significance was determined by multiple comparison tests using the analysis of variance (ANOVA).

### Ethics statement

This study was approved by Animal Care and Use Committee of Aoyama Gakuin University (A9/2020) and carried out according to the Aoyama Gakuin University Animal Care and Use Guideline.

## Supplementary Information

Supplementary Figure S1.
