# Supplementary material for: Loss of αklotho causes reduced motor ability and short lifespan in zebrafish
Source: Sci Rep. 2021 Jul 23;11:15090. doi: 10.1038/s41598-021-93909-y (PMC8302672; doi:10.1038/s41598-021-93909-y)
Supplement: Supplementary file 1 — Supplementary Figure S1. [file 41598_2021_93909_MOESM1_ESM.pdf]

Loss of αklotho causes reduced motor ability and short lifespan in zebrafish

Yurie Ogura, Kota Ujibe, Ryoji Kaneko, Yuma Wakamatsu and Hiromi Hirata*

Department of Chemistry and Biological Science, College of Science and Engineering, Aoyama Gakuin University, Sagamihara 252-5258, Japan

***Correspondence:** Hiromi Hirata hihirata@chem.aoyama.ac.jp

Number of supplementary figure: 1

Figure S1. The αKlotho is conserved in vertebrates.

Multiple amino acid sequence alignment of the αKlotho protein. Black and grey colors respectively indicate the completely and highly conserved amino acid residues among vertebrates. The N-terminal signal peptide is highlighted by an orange box. The first and the second putative glycosidase domains are indicated by blue and magenta boxes, respectively. The C-terminal transmembrane domain is highlighted by a green box. Yellow circles indicate putative N-linked glycosylation sites. Two red arrows indicate the position of the frameshift mutation (S179frameshift) published in a recent zebrafish *kl* mutant study (allele name not assigned) and the position of the Y306X protein truncation in our allele ($kl^{sa18644}$). Following NCBI data were used: human (*H. sapines*) NP_004786; rhesus monkey (*Macaca mulatta*) XP_001101127; rat (*Rattus norvegicus*) NP_112626; mouse (*Mus musculus*) NP_038851; chicken (*Gallus gallus*) XP_417105; tropical clawed frog (*Xenopus tropicalis*) XP_002934067; zebrafish (*Danio rerio*) XP_021335093.

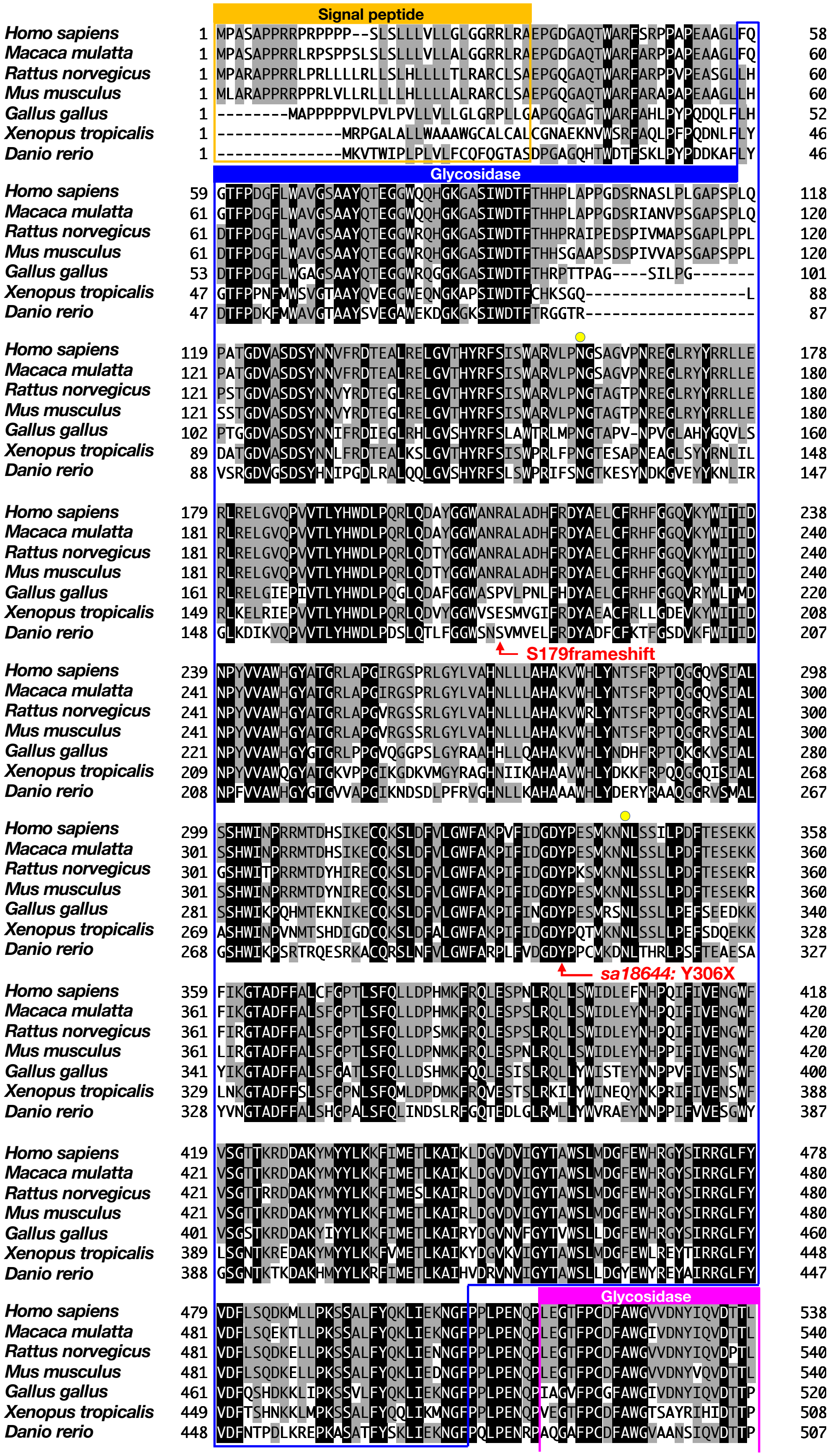

Ogura Y et al. Supplementary Fig. S1

```
                                                    Glycosidase
Homo sapiens          539 SQFTDLNVYLWDVHHSKRLIKVDGVVTK--KRKSYCVDFAAIQPQIALLQEMHVTHFRFS  596
Macaca mulatta        541 SQFTDLNVYLWDVHHSKRLIKVDGVVTK--KRKSYCVDFAAIQPQITLLQEMHVTHFRFS  598
Rattus norvegicus     541 SQFTDPNVYLWDVHHSKRLIKVDGVVAK--KRKPYCVDFSAIRPQITLLREMRVTHFRFS  598
Mus musculus          541 SQFTDPNVYLWDVHHSKRLIKVDGVVAK--KRKPYCVDFSAIRPQITLLREMRVTHFRFS  598
Gallus gallus         521 AQFLDPNVYVWDVHQTKKLIKVDGVFTS--QRKHHCVDFAAIRLQISLLQEMHVTHFHFS  578
Xenopus tropicalis    509 SQFNDPNVYVWDMNKTKGLTKVEGITVP--KRKTQCVDFASIRQQISMLREIHITHFYFA  566
Danio rerio           508 TQFTDTNVYVWNISGNGELKKLPGLQAPHLRRTPHCADYGSIRQQVSDLLRMQVSHFHFS  567

Homo sapiens          597 LDWALILPLGNQSQVNHTILQYYRCMASELVRVNITPVVALWQPMAPNQGLPRLLARQGA  656
Macaca mulatta        599 LDWALILPLGNQSQVNHTILQYYRCMVSELVRVNITPVVALWQPVAPNQGLPRLLARQGA  658
Rattus norvegicus     599 LDWALILPLGNQTQVNRTVLHFYRCMVSELVHANITPVVALWQPATPHQGLPHALAKHGA  658
Mus musculus          599 LDWALILPLGNQTQVNHTVLHFYRCMISELVHANITPVVALWQPAAPHQGLPHALAKHGA  658
Gallus gallus         579 LKWSSVLPLGNLSLINHTLVHYYQCFASELLRVNITPVVALWQPMAENQELPTSLAKFGA  638
Xenopus tropicalis    567 LKWAAILPLGNLSLIHHKVLHYYQCFVSELVRVNITPVVALWQPLAENQGLPIDLAKNGG  626
Danio rerio           568 LNWSSIVPTGHVSDANETLLRYYYCFVSELQKVNITPVVTLWHHTGKLSSLPAPMEASDG  627

Homo sapiens          657 WENPYTALAFAEYARLCFQELGHHVKLWITMNEPYTRNMTYSAGHNLLKAHALAWHVYNE  716
Macaca mulatta        659 WENPYTALAFAEYARLCFQELGHHVKLWITMNEPYTRNMTYNAGHNLLKAHALAWHVYNE  718
Rattus norvegicus     659 WENPHTALAFADYANLCFEELGHWVKFWITINEPNSRNMTYRAGHHLLKAHALAWHLYDD  718
Mus musculus          659 WENPHTALAFADYANLCFKELGHWVNLWITMNEPNTRNMTYRAGHHLLRAHALAWHLYDD  718
Gallus gallus         639 WENSETVQAFVEYAKFCFASLGDHVKFWITMNEPSVKNLTYTAGHNLLRAHAKAWHLYDK  698
Xenopus tropicalis    627 WVNYHTVSAFVEYARLCFKELGNYVGMWITMNEPSMRNLTYAAGHNLLKAHALAWHLYDR  686
Danio rerio           628 WQSEKTVQAFVDYARLCFQRLGAHVKLWITLNEPNDEDLEYTVGHQLLRAHALAWHVYDR  687

Homo sapiens          717 KFRHAQNGKISIALQADWIEPACPFSQKDKEVAERVLEFDIGWLAEPIFGSGDYPWVMRD  776
Macaca mulatta        719 KFRHAQNGKISIALQADWIEPACPFSQKDKEVAERVLEFDIGWLAEPIFGSGDYPWVMRD  778
Rattus norvegicus     719 KFRAAQKGKISIALQVDWIEPACPFSQKDKEVAERVLEFDVGWLAEPIFGSGDYPHVMRE  778
Mus musculus          719 KFRAAQKGKISIALQADWIEPACPFSQNDKEVAERVLEFDIGWLAEPIFGSGDYPRVMRD  778
Gallus gallus         699 EFRRSQKGKISIALQADWVEPACPFSRNDQEVADRILEFDIGWLAEPIFGSGDYPHMMRA  758
Xenopus tropicalis    687 DFRKAQKGQISIAVQADWVEPASPFSKNDKETSRRILEFEIGRLSDPIFLSGDYPKVMRD  746
Danio rerio           688 EFRKAQGGKASLVLHMDWVEPAFSFNREDVAPADRVLDFRVGWFAEPIFGKGDYPAVMRS  747

Homo sapiens          777 WLNQRN-----NFLLPYFTEDEKKLIQGTFDFLALSHYTTILVDSEKEDPIKYNDYLEVQ  831
Macaca mulatta        779 WLNQRN-----NFLLPYFTEDEKKLIQGTFDFLALSHYTTILVDSEKEDPIKYNDYLEVQ  833
Rattus norvegicus     779 WLNQKN-----NFLLPYFTEDEKKLIRGSFDFLALSHYTTILVDWEKEDPIKYNDYLEVQ  833
Mus musculus          779 WLNQKN-----NFLLPYFTEDEKKLVRGSFDFLAVSHYTTILVDWEKEDPMKYNDYLEVQ  833
Gallus gallus         759 WLHQRNSVDLYNFHLPSFSEDEKKLIQGSFDFFALSHYTTILVGWEKEDALKYDHYLEVQ  818
Xenopus tropicalis    747 WLAPRNNLDVFEFYLPSFTEEEKNLIQGTFDFFALSHFTTELVQWEKEDVAKYDHRLEVQ  806
Danio rerio           748 WLQQRNTIDLFNYHLPTFSEEDRLLVKGTYDFFAISHFTTSMVYDGVEDKYTFKDKLQVQ  807

Homo sapiens          832 EMTDITWLNSPSQVA-VVPWGLRKVLNWLKFKYGDLPMYIISNGIDDGLHAEDDQLRVYY  890
Macaca mulatta        834 EMTDITWLNSPSQVA-VVPWGLRKVLNWLKFKYGDLPMYIISNGIDDGLHAEDDQLRVYY  892
Rattus norvegicus     834 EMTDITWLNSPNQVA-VVPWGLRKALNWLRFKYGDLPMFVTANGIDDDPHAEQDSLRMYY  892
Mus musculus          834 EMTDITWLNSPSQVA-VVPWGLRKVLNWLRFKYGDLPMYVTANGIDDDPHAEQDSLRIYY  892
Gallus gallus         819 MISDITWLHSPSRAA-VVPWGLRKVLRWVKSKYGDVPVYVMANGIDDDQNMVHDKLRVYY  877
Xenopus tropicalis    807 FITDSTWVHSPNKYA-VVPWGLRKVLNWVKSKYGDVPIYILANGIDDAHSPMQDKLRVYY  865
Danio rerio           808 LISDVTWIMSPRRNSPVVPWGLRKALNWVNSRYKGVPIYVMANGVQEDTARFRDSLRSYY  867

Homo sapiens          891 MQNYINEALKAHILDGINLCGYFAYSFNDRTAPRFGLYRYAADQFEPKASMKHYRKIIDS  950
Macaca mulatta        893 MQNYINEALKAHILDGINLCGYFAYSFNDRTAPRFGLYRFAADQFEPKPSMKHYRKIIDS  952
Rattus norvegicus     893 IKNYVNEALKAYVLDGINLCGYFAYSLSDRSVPKSGFYRYAANQFEPKPSIKHYRKIIDN  952
Mus musculus          893 IKNYVNEALKAYVLDDINLCGYFAYSLSDRSAPKSGFYRYAANQFEPKPSMKHYRKIIDS  952
Gallus gallus         878 IQNYINEALKAYALDNVNLQGYFVYSFNDKTAPKYGLYSYAANQYEPKPSMKHYREIIDN  937
Xenopus tropicalis    866 LQNYINEALKAILHDGVNLRGYFAYSFNDRMDPRYGLYAYAANRFAPKLSMKHYQEIIDN  925
Danio rerio           868 LYNYVNEALKAYMLDAVNLKGYFAYAFSDQRDPGFGMYGHVQEEVISKSSLGHYKNIIRH  927

                                                   Membrane
Homo sapiens          951 NGFPGPETLERFCPEEFTVCTECSFFHTRKSLLAFIAFLFFASIISLSLIFYYSKKGRRS 1010
Macaca mulatta        953 NGFPGPETLEKFCPEEFTVCTECSFFHTRKPLVAFIAFLFFAFIVSLSLIFYYSKKGRRR 1012
Rattus norvegicus     953 NGFLGSGTLGRFCPEEYTVCTGCGFFQTRKSLLAFISFLVFAFVTSLALIYYYSKKGRRR 1012
Mus musculus          953 NGFLGSGTLGRFCPEEYTVCTECGFFQTRKSLLVFISFLVFTFIISLALIFHYSKKGQRS 1012
Gallus gallus         938 NGFPGPDTAEVLCPEEAAMCPECHFFRTRKSLFAFISFIFVAFIVTIFCIMYYSKRAERR  997
Xenopus tropicalis    926 NGFPNPEMPAVSCPVELVPCSDCHFFQTRKYLLAFVAFIFIVLIVSVFMITYYSRKGKRR  985
Danio rerio           928 NGFPAPSTSQHQCPHAPAQGSG-RYVLTKKPVVGFLSLVSSCMLITMCLVIYYAFKRHKL  986

Homo sapiens         1011 YK--                                                         1012
Macaca mulatta       1013 YK--                                                         1014
Rattus norvegicus    1013 YK--                                                         1014
Mus musculus         1013 YK--                                                         1014
Gallus gallus         998 YK--                                                          999
Xenopus tropicalis    986 YK--                                                          987
Danio rerio           987 TTKK                                                          990
```

Ogura Y et al. Supplementary Fig. S1
